# Evolutionary divergence of phytochrome protein function in *Zea mays* PIF3 signaling

**DOI:** 10.1093/jxb/erw217

**Published:** 2016-06-04

**Authors:** Indrajit Kumar, Kankshita Swaminathan, Karen Hudson, Matthew E. Hudson

**Affiliations:** ^1^Department of Crop Sciences, University of Illinois at Urbana-Champaign, Urbana, IL 61801, USA; ^2^Physiology and Molecular Plant Biology, University of Illinois at Urbana-Champaign, IL 61801, USA; ^3^USDA-ARS Crop Production and Pest Control Research Unit, 915 West State Street, West Lafayette, IN 47907, USA

**Keywords:** Arabidopsis, bHLH, PIF, maize, phyB, phytochrome, subfunctionalization.

## Abstract

Two maize phytochrome-interacting factor (PIF) basic helix-loop-helix (bHLH) family members, ZmPIF3.1 and ZmPIF3.2, were identified, cloned and expressed *in vitro* to investigate light-signaling interactions. A phylogenetic analysis of sequences of the maize bHLH transcription factor gene family revealed the extent of the PIF family, and a total of seven predicted PIF-encoding genes were identified from genes encoding bHLH family VIIa/b proteins in the maize genome. To investigate the role of maize PIFs in phytochrome signaling, full-length cDNAs for phytochromes *PhyA2*, *PhyB1*, *PhyB2* and *PhyC1* from maize were cloned and expressed *in vitro* as chromophorylated holophytochromes. We showed that ZmPIF3.1 and ZmPIF3.2 interact specifically with the Pfr form of maize holophytochrome B1 (ZmphyB1), showing no detectable affinity for the Pr form. Maize holophytochrome B2 (ZmphyB2) showed no detectable binding affinity for PIFs in either Pr or Pfr forms, but phyB Pfr from Arabidopsis interacted with ZmPIF3.1 similarly to ZmphyB1 Pfr. We conclude that subfunctionalization at the protein-protein interaction level has altered the role of phyB2 relative to that of phyB1 in maize. Since the *phyB2* mutant shows photomorphogenic defects, we conclude that maize phyB2 is an active photoreceptor, without the binding of PIF3 seen in other phyB family proteins.

## Highlight

phyB and PIF3 share a common protein-protein signaling mechanism in maize and Arabidopsis. However, phyB1 and phyB2 in maize do not share this mechanism, despite being closely related, indicating recent evolutionary divergence.

## Introduction

Phytochromes are involved in perceiving red (R) and far-red (FR) light signals that are essential for growth and development in plants. The phytochrome family in Arabidopsis, the best characterized plant photomorphogenic system, contains five members: *PHYA*, *PHYB*, *PHYC*, *PHYD* and *PHYE* (Sharrock and Quail, 1989; Clack *et al.*, 1994). Phytochromes toggle between two interconvertible forms, Pfr (the FR absorbing, active form) and Pr (R absorbing, inactive form) in response to light signals. Active phytochrome in the Pfr form transduces downstream signaling in part by interacting with bHLH transcription factors known as phytochrome interacting factors (PIFs) (Ni *et al.*, 1998, 1999; Monte *et al.*, 2007; Shin *et al.*, 2007; Leivar *et al.*, 2008). This interaction leads to phosphorylation and subsequent proteasome-mediated degradation of PIFs (Ni *et al.*, 2014). This, in turn, results in a change in the gene expression profile that is part of the program of de-etiolation and photomorphogenesis (Leivar *et al.*, 2012). About 10% of the Arabidopsis transcriptome is under the control of phytochromes during de-etiolation (Quail, 2002). Phytochrome signaling has been largely investigated in model systems, and is not well characterized in monocots, including agronomically and economically important crops such as maize, rice, wheat and sorghum. Phytochromes have, however, been shown to control several agronomically important traits such as axillary stem (tiller) number in sorghum and rice (Foster *et al.*, 1994; Kong *et al.*, 2004; Kebrom *et al.*, 2006) and stand density in maize (Maddonni *et al.*, 2002; Fellner *et al.*, 2003).

The genome of maize (*Zea mays* L.), a globally important crop, has been sequenced and annotated, and mutants in phytochrome genes have been identified (Sheehan *et al.*, 2007). While in Arabidopsis *PHYB*, *PHYD* and *PHYE* are closely related and represent an expanded *PHYB* gene family, the genomes of monocots characterized so far contain one or more sets of the three phytochrome genes *PhyA*, *PhyB* and *PhyC* (Mathews and Sharrock, 1996, 1997). Phytochrome A, B and C genes are present in single copies in rice and sorghum (Takano *et al.*, 2001), however, due to a recent genome duplication event, maize contains two homeolog pairs of each of the three gene families, namely *PhyA1* and *PhyA2*; *PhyB1* and *PhyB2*; *PhyC1* and *PhyC2* (Childs *et al.*, 1997; Christensen and Quail, 1989; Basu *et al.*, 2000). Monocots and dicots have evolved independently since ~140–200 Mya, thus it is possible that the biomolecular functions of monocot and dicot phytochromes may not be totally conserved (Sheehan *et al.*, 2004). In fact, studies have indicated unique roles of Arabidopsis and rice phytochromes, suggesting fundamental differences between monocot and dicot phytochrome-mediated pathways (Takano *et al.*, 2001, 2005; Franklin and Whitelam, 2004). This hypothesis is also supported by the fact that the maize genome lacks *PhyD* and *PhyE* counterparts and at the same time possesses two homeologs each of *PhyA*, *PhyB* and *PhyC*.

Acting as transcriptional activators or repressors, bHLH proteins have been found to be involved in variety of plant gene-regulatory functions including phytochrome signaling, hormone signaling, stress responses and control of secondary metabolic pathways (Castillon *et al.*, 2007; Feller *et al.*, 2011). Many plant bHLH proteins have been shown to have preferential recognition and binding to the G-box motif, a subset of the E-box motif with sequence 5′-CACGTG-3′ (Martinez-Garcia *et al.*, 2000; Huq and Quail, 2002; Huq *et al.*, 2004). The PIFs belong to one of the plant bHLH subfamilies (VIIa/b) and are arguably the best characterized phytochrome interacting proteins, performing key roles in phytochrome-mediated photomorphogenesis in Arabidopsis (Leivar and Monte, 2014). Here we investigate the PIF family of proteins in maize, and test the extent to which phytochrome signaling pathways are conserved between Arabidopsis and a monocot crop system.

## Materials and methods

### bHLH gene search

The maize predicted protein dataset [ZmB73_5b_FGS_translations.fasta (filtered gene set)] was obtained from www.maizesequence.org (Maize genome sequence release 5b). Putative bHLH sequences were identified by using a hidden Markov model search (hmmsearch v. 3.0; http://hmmer.janelia.org/) (Eddy, 1998) across the set of maize predicted proteins. The PFAM HLH hidden Markov model (PF000010; http://pfam.sanger.ac.uk) was used for this search using default hmmsearch settings. A total of 312 hits containing putative HLH domains were identified. A total of 204 hits representing unique loci were selected on the basis of presence of a complete HLH domain or longer protein sequence. Hits with partial HLH domains were manually curated to identify mispredictions of splice sites. Seven hits with incomplete HLH domains (GRMZM2G137380, GRMZM2G138454, GRMZM2G175955, GRMZM2G317317, GRMZM2G452996, GRMZM5G896413 and GRMZM2G100313) were discarded and finally 197 entries were used for further analysis. Orthologous gene information was obtained from www.phytozome.org (Zmays_181_annotation_info.txt).

### BLAST, sequence alignment and protein domain/motif analysis

BLASTs were performed using tools provided at NCBI, http://www.arabidopsis.org/ and http://www.maizesequence.org. Alignments were performed using the MAFFT and/or MUSCLE tools in Geneious Pro v5.6.5 (Biomatters Ltd., Auckland, New Zealand). For identification of domains and motifs in protein sequences, Pfam (http://pfam.sanger.ac.uk/) and prosite (http://prosite.expasy.org/) scan tools were used. Screening of conserved motifs outside the bHLH domain was done using the program hmmsearch v3.0 from the hmmer package (Eddy, 1998) using a hidden Markov model generated for each motif as follows: alignments of 26 conserved motifs were obtained from the supplementary material of Pires and Dolan (2010); hidden Markov models for motif alignments were generated by hmmbuild 2.3.2 from the hmmer package, and each model was screened across the full-length proteins of the 197 bHLH maize genes again using hmmsearch as above; an e‐value cut off of 1 was used for the search; graphic representations of domains were created by DOG v2.0 (Ren *et al.*, 2009).

### Sequence alignment and phylogenetic analysis

Multiple alignments were created using MAFFT v6 (Windows) using the –auto, ‐‐reorder options. Visualization and editing of alignments was performed using Geneious Pro v5.06 (Biomatters Ltd, Auckland, New Zealand) and MEGA v5.05. Identification of conserved residues and E‐box/G‐box binding motifs in the alignment was carried out using Geneious Pro v5.06. The bHLH sequences used for our analysis and present in the alignment were renamed as ZmbHLHXXX as per Bailey *et al.* (2003) and Heim *et al.* (2003). Maximum likelihood phylogenetic analysis of the bHLH sequence alignment was performed using RAxMLHPC‐MPI v 7.2.6 (Stamatakis, 2006) using the JTT model of amino acid substitutions and gamma distribution parameters estimated by the software. Regions with gaps in the alignment were removed. 1000 rapid bootstraps were performed. Phylogenetic trees were visualized and edited in MEGA v5.05. For classification of ZmbHLHs into subfamilies, representative bHLH sequences for each subfamily from Arabidopsis, rice and *Physcomitrella patens* (Supplementary Table S1 at *JXB* online) were added to the alignment and a maximum likelihood tree was created as above.

### Cloning and site directed mutagenesis

Maize *PhyB1* (GRMZM2G124532) CDS sequence was amplified as N-terminal (1–2348bp) and C-terminal (1942–3534bp) parts. The N-terminal region was obtained by RT-PCR using MH289 (5′-ACGGGGCCGCCTAATCCAGA-3′) and MH287 (5′-CAACTGTCGCCTCAGATTCCTTGAAGATAAGATCA-3′) primers. The C-terminal fragment was amplified by RT-PCR using MH177 (5′-GATACATATGCTTAGTTCCGTAGCAAGA GAGAT-3′) and MH178 (5′-GAAAGGATCCGGGTCAGCACG GATCTTAAC-3′) primers. The N-terminal and C-terminal fragments were joined by the transformation-assisted recombination method in yeast (Larionov *et al.*, 1996).

*ZmPhyB2* CDS (GRMZM2G092174) was also amplified in two parts: an N-terminal fragment (1–2068bp) and a C-terminal fragment (1976–3501bp). The N-terminal fragment was amplified by RT-PCR using MH349 (zPB2-F) (5′-ATGGCGTCGGACAGTCGCCCCC-3′) and MH354 (zPB2-2.07Rev) (5′-GGCCTGTCAACTCGGCAATCTTTGCATTC CAC-3′) and TAKARA Ex Taq DNA polymerase (Clontech Labs, CA, USA). Reverse transcription was performed as described above with MH350 (zPB2-R-RT) (5′-GGTGTCAGCACGGATCT TAACATATCAGAC-3′). The C-terminal fragment was amplified from maize cDNA library using MH384 (zPB2 1976F) (5′-CGATTGATAGAGACAGCAACAGTACCCATA-3′) and zPB2 Sp 3’Rev (5′-TTAACATATCAGCTGATTTTCTC TACCAGCTGC-3′). The N-terminal and C-terminal fragments were joined by the transformation-assisted recombination method in yeast (Larionov *et al.*, 1996).

*ZmPhyA2* CDS (GRMZM2G181028) was also amplified in two parts: an N-terminal fragment (1–1710bp) and a C-terminal fragment (1517–3396bp). The N-terminal fragment was amplified by RT-PCR using MH376 zPhyA-5F (5′- ATGTCTTCCTTGAGGCCTGCCCAG-3′) and MH404 zPA1sp-1720 Rev (5′-TGGCAAACTCTTCATCTTGACA ACCTCG-3′) and TAKARA Ex Taq DNA polymerase (Clontech Labs, CA, USA). The C-terminal fragment was amplified from the maize cDNA library using MH403 zPA1sp-1517-F (5′-ACATGATCTGTGGAATGGCAGTGGCT-3′), MH377 zPA1-3R (5′-TCAATGTCCAGCTGCTGAAGGAGCA-3′) primers. The N-terminal and C-terminal fragments were joined by transformation-assisted recombination method in yeast (Larionov *et al.*, 1996).

*ZmPhyC1* CDS (GRMZM2G057935) was amplified in two parts: an N-terminal fragment (1–1922bp) and a C-terminal fragment (1828–3408bp). The N-terminal fragment was amplified by RT-PCR using MH380 zPC1-5F (5′-ATGTCGTTGCCGTCGAACAACC-3′) and MH383 zPC1-1923-R (5′-TTACCGGCAATGTCGACAGCCAA-3′) and TAKARA Ex Taq DNA polymerase (Clontech Labs, CA, USA). The C-terminal fragment was also amplified by RT-PCR using MH382 zPC1-1855-F (5′-CAGGGGCTACTTGAACTG AGAACAGTT-3′) and MH381 zPC1-3R (5′-TCAGAATTTACT CGTCGAAGGCTTGGAC-3′) primers. The N-terminal and C-terminal regions were merged using the restriction site SalI present in the overlapping region (1828–1922bp).

*ZmPi3f.2* (GRMZM2G387528_T02) CDS was amplified from *Zea mays* cv. B73 cDNA library. We found an additional start codon, 93 nucleotides upstream of the reported start site in the cDNA thus potentially coding for additional 31 amino acids. We used the longer version in this study. *Pfu* Ultra II Fusion HS DNA Polymerase (Stratagene, CA, USA, Cat # 600674) was used for PCR amplification with zPIFIII Ext1 HindIIIF (5′-AGCAAAGCTTCATGAGCAAGGAGCCCTGCTGCT-3′) and MH256 (5′-CGACGAATTCTCATGTTTCAGCCTCAT TTCT-3′) primers.

*ZmPif3.1* (GRMZM2G115960) CDS was amplified from the same maize cDNA library using GRMZM2G115960-T03-NdeI-F (5′-CATATGTCCGACAGCAACGACTTC-3′) and GRMZM2G115960-T03-Rev (5′-GATTACTGGCGAAGATCTCT TCATC-3′) primers and TAKARA Ex Taq DNA polymerase (Clontech Labs, CA, USA).

Targeted Y2H Phy-CTD bait constructs were created by re-amplification using primer combinations (Table 1) followed by subcloning in the pGBKT7 vector.

**Table 1.** Primers used in phy-CTD subcloning

Construct	Region (nt)	Forward Primer	Reverse Primer
ZmPhyB1-CTD	1933–3534	5′-CTAGCATATGATAAATGAGCTTAGTTCCGTAG-3′	5′-GCTAGTTATTGCTCAGCGGT-3′ (Vector Specific)
ZmPhyB2-CTD	1936–3501	5′-GATTATTAATGGGATAAATGAGCTTAGCTCTG-3′	
ZmPhyA2-CTD	1831–3410	5′-TCGACATATGCTTGATGGGCTTGCTGAATTGC-3′	
ZmPhyC1-CTD	1831–3435	5′-CGATCATATGGGGCTACTTGAACTGAGAACAG-3′	

Immunoprecipitation plasmid constructs were created in the pET17b vector (Novagen; cat. no. 69663-3) except *AtPHYB* where pET3a (Novagen; cat. no. 69418-3) was used. Y2H constructs were created in pGBKT7 (Clontech, CA; cat. no. PT3248-5) and pGADT7 (Clontech, CA; cat. no. PT3249-5) vectors. *ZmPhyB1-4B2* (F111L, L164Y, P341Q and S707C) and *ZmPhyB2-4B1* (L113F, Y166L, Q343P and C709S) constructs were created using the method described in QuickChange Multi Site-Directed Mutagenesis Kit (Stratagene, CA, USA; cat. no. 200514).

### Yeast two-hybrid screening

The C‐terminal domain of ZmPhyB1 was used as bait for the yeast two-hybrid (Y2H) screening. Yeast strain YRG2 (Stratagene, La Jolla; cat. no. 240060) competent cell preparation and transformation with GBD:*ZmPhyB1*‐CTD construct was performed as per the standard protocol suggested by Frozen‐EZ Yeast Transformation II Kit (Zymo Research, Irvine, CA, USA). Y2H screening was performed as per Matchmaker Gold Yeast Two‐Hybrid System (Clontech, CA; cat. no. PT4084). An in‐house size‐fractionated cDNA library was made from etiolated *Zea mays* cv. B73 seedlings using the Stratagene Lambda Hybri‐Zap vector (Stratagene, La Jolla, CA) and was used as screening prey. A positive control was created by co‐transforming BD:*AtPHYB*‐CTD and GAD:*AtPIF3* constructs. Negative controls were created by co‐transforming BD:*ZmPhyB1*‐CTD and empty GAD vector (pGADT7 or pGAD424).

### Immunoprecipitation assay

Bait and prey proteins were synthesized *in vitro* using ^35^S‐Methionine in the TNT T7 Quick Coupled Transcription/Translation System (Promega, Madison, WI; cat. no. L1170). For each immunoprecipitation, 15 μl of magnetic Dynabeads Protein A (Invitrogen, CA, USA) was used. 30 μl of beads was washed with 0.1ml of 1× binding buffer (PBS buffer pH 7.25, 0.1% Tergitol NP‐40 (Sigma), 1mM EDTA, 0.1mg/ml BSA, 1 cOmplete Mini Protease Inhibitor (Roche Applied Science, IN, USA) per 10ml of PBS buffer). Antibody binding was performed by incubating beads with 100 μl of 1× binding buffer with 3 μg of Anti‐GAD antibodies (Santacruz Biotechnology, CA; cat. no. 1663) and rotating at 4 °C for 1.5h. Supernatant was removed using Dynal MPC magnetic stand (Invitrogen, CA) and beads were resuspended in 24 μl of bait TNT and 96 μl of 1× binding buffer (1:5 ratio) and again rotated at 4 °C for 3–4h. Phytochrome TNT was precleared and holophytochrome was prepared by adding 5 μl of prewashed Protein A Dynabeads and 40–60 μM of bacterial Phycocyanobilin (Frontier Scientific, Logan, UT; cat. no. P14137) to 95 μl of phytochrome TNT and 95 μl of 2× binding buffer and rotated at 4 °C for 1h in darkness. Using a magnetic stand, holophytochrome was collected and divided (~95 μl each) into two 1.5ml microfuge tubes. Both tubes were incubated horizontally on ice under LED red light (1.5 μM m^−2^ s^−1^) for 10min to convert phytochrome to the Pfr form. One tube (Pfr) was removed and stored on ice in darkness and the other tube (Pr) was treated for 5min of LED far‐red light (1 μM m^−2^ s^−1^). Bait beads were gently resuspended and divided equally into two 1.5ml microfuge tubes. Bait beads were captured and supernatant was removed by using a magnetic stand. Pr and Pfr holophytochromes were added to one of each bait bead-containing tube, mixed gently and rotated at 4 °C for 4h in darkness. The pulldown was ended by collecting beads with the magnetic stands and washing the beads once with 0.5ml 1× binding buffer and twice with 0.5ml 1× wash buffer (binding buffer without BSA). Before the last wash, the beads were transferred to a new tube. Bead bound proteins were eluted by resuspending beads into 30 μl of 2× Laemmli’s buffer (with DTT) and heating at 65 °C for 5min. The protein samples were run on 4–20% gradient SDS-PAGE (Bio‐Rad, Hercules, CA). The gel was fixed in acetic acid:methanol:glycerol (7:7:10% solution) by shaking for 10min and then dried in a Bio‐Rad 583 gel dryer for 1.5–2h. The dried gel was then exposed to a phosphor screen overnight and scanned using a Storm 840 Phosphorimager (Amersham). All light-sensitive steps (until denaturation of proteins) were performed in a dark room under green safe light. The spectrum of green safe light, red light and far red light used is shown in Supplementary Fig. S1, measured using a Stellarnet EPP200 spectroradiometer.

## Results

### A PIF3-like protein is a primary PHYB1 interacting factor in maize

**Fig 1.**
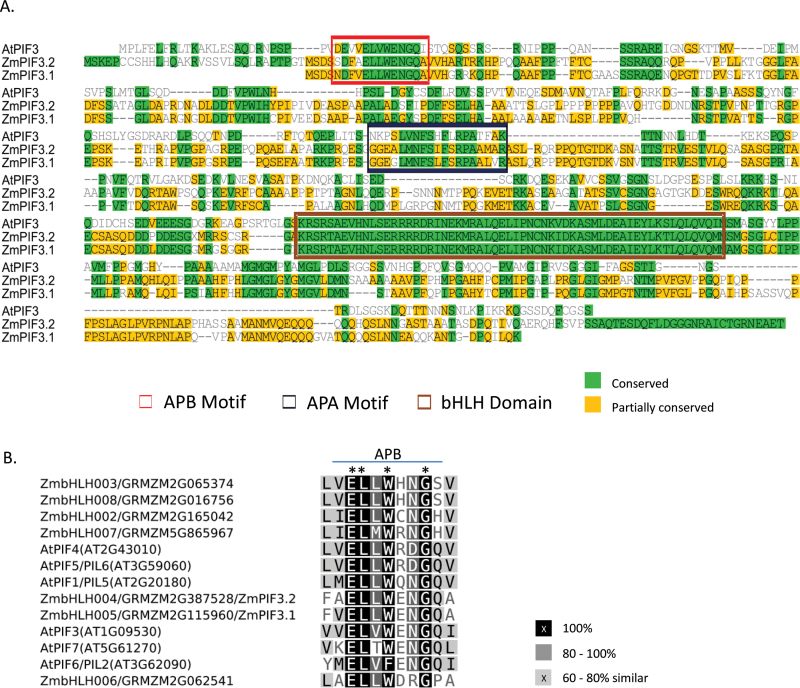
Sequence conservation of Arabidopsis and maize PIFs. (A) Multiple peptide sequence alignment of Arabidopsis PIF3 (AtPIF3), maize PIF3.1 (ZmPIF3.1, otherwise ZmbHLH005, GRMZM2G115960) and maize PIF3.2 (ZmPIF3.2, ZmbHLH004, GRMZM2G387528) showing the conserved bHLH domain, APB motif and APA motif. (B) Partial alignment of seven maize bHLH peptide sequences from subfamily VIIa/b and Arabidopsis PIFs (AtPIFs) showing the highly conserved active phyB binding motif (APB; also known as Motif 14) used to generate a hidden Markov model. (This figure is available in colour at *JXB* online.)

The C-terminal domain of phytochromes interacts with phytocrome signaling partners and has been used as bait in Y2H screens to identify interacting proteins (Ni *et al.*, 1998, 1999). PIF3 was the first phytochrome interacting factor identified in Arabidopsis. In a similar approach, we used the C-terminal domain of maize PHYB1 (647–1161 aa, PHYB1-CTD) as bait to screen a size-fractionated maize cDNA Y2H library from etiolated seedlings. This Y2H screen identified a bHLH domain-containing protein (GRMZM2G387528) as a primary PHYB1-CTD interacting candidate. The bHLH domain of GRMZM2G387528 is closely related to that of Arabidopsis PIF3 (AT1G09530.1) (Fig. 1A). This gene is located on maize chromosome 8; the region has been defined to be in maize subgenome 2 and we thus refer to it as *ZmPif3.2* (the ‘2’ indicating subgenome number). Like most maize genes, *ZmPif3.2* has a homeolog, in this case on subgenome 1, on chromosome 3 (GRMZM2G115960) which we henceforth refer to as *ZmPif3.1*. ZmPIF3.2 shares a very high degree of protein sequence identity with ZmPIF3.1, and it also interacts with PHYB1-CTD in a targeted Y2H assay (Supplementary Fig. S2). Alignment of ZmPIF3.1, ZmPIF3.2 and AtPIF3 protein sequences indicates conserved bHLH sequences along with the APB (active phyB binding) motif and APA (active phyA binding) motif. These motifs indicate likely evolutionary conservation of ZmPIF3.2 binding to phyB and phyA, respectively (Fig. 1A).

### The maize bHLH subfamily VIIa/b encodes at least 15 members, including at least seven PIF-like proteins

In order to investigate the PIF gene family in maize, we performed a hidden Markov model (HMM) search using the PFAM HLH HMM (PF00010) on the maize protein dataset [ZmB73_5b_FGS (filtered gene set); www.maizesequence.org] and obtained 312 protein sequences containing putative bHLH domains. A multiple sequence alignment was then created using the 197 unique, complete bHLH-domain amino acid sequences. Maximum likelihood phylogenetic analysis was then used to create a phylogeny of the bHLH domains from the alignment, in order to assign subfamily membership (Supplementary Figs S3, S4).

In general, the amino acid sequence outside of the core bHLH domain is too divergent for accurate multi-sequence alignment. However, at least 28 conserved non-bHLH motifs have been reported in plant bHLH proteins and members of the same bHLH subfamilies share highly conserved motifs (Pires and Dolan, 2010). Searches with HMMs generated using the sequence alignments of these conserved motifs were performed on 197 full-length putative bHLH protein sequences from maize. Except for motif 15, the rest of the 27 motifs were found in one or more of the ZmbHLH proteins. A complete list of maize bHLH genes with non-bHLH motif information is provided in Supplementary Table S2. Most of the maize bHLH subfamilies shared common motifs, providing additional support for the phylogenetic classification of ZmbHLH members. Motif 14, typically present in members of the bHLH subfamily VIIa/b, corresponds to the active phytochrome binding (APB) motif. In Arabidopsis, the APB motif is crucial for the binding of PIF family members to phytochrome B (Khanna *et al.*, 2004). A total of 15 members of the VIIa/b subfamily were identified in our phylogenetic analysis of bHLH domains in maize. Seven ZmbHLH VIIa/b proteins (those in the range ZmbHLH002–008), including ZmPIF3.1 (ZmbHLH005; GRMZM2G115960) and ZmPIF3.2 (ZmbHLH004; GRMZM2G387528) had a region outside the bHLH domain showing strong similarity to motif 14/APB, and are thus strong candidates for PIFs (Fig. 1B). According to previous reports, bHLHs of subfamily VIIa/b also contain motifs 15 and 16 (Pires and Dolan, 2010); however we found maize bHLHs to completely lack motif 15. The putative PIFs instead contain motif 23 that is typically found in subfamily XII. The remaining members of the subfamily VIIa/b did not contain detectable similarity to any of the 28 previously described secondary motifs. More details and annotations for the seven putative maize PIFs are included in Supplementary Table S3.

### The PIF3.1 and PIF3.2 proteins interact with phyB1 holophytochrome in the Pfr form *in vitro*

**Fig 2.**
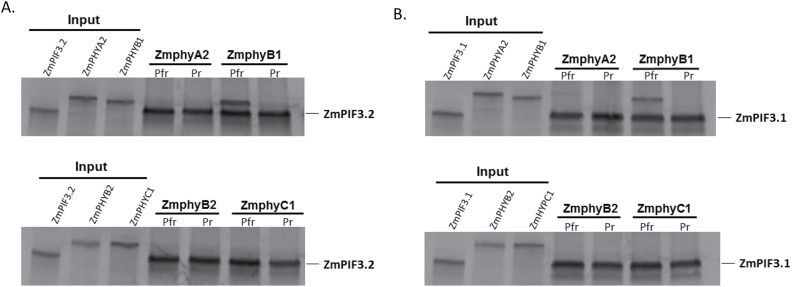
Phytochrome:PIF3 interaction studies. Gel autoradiographs from *in vitro* co-immunoprecipitation studies for ^35^S-labeled maize phytochromes (Zmphy) in the Pr (inactive) and Pfr (active) forms co-precipitated with ^35^S-labeled maize PIF3 proteins bound to a solid matrix. (A) ZmphyB1 (Pfr) shows interaction with PIF3.2; however ZmphyA2 (Pfr) does not interact detectably in this experiment (upper panel). No interaction was detected between either ZmphyB2 (Pfr) or ZmphyC1 (Pfr) and PIF3.2 (lower panel). (B) ZmphyB1(Pfr) also interacts with PIF3.1; any interaction with phyA2(Pfr) is weak (upper panel). Neither phyB2 nor phyC1 (Pfr) interacts with PIF3.1 (lower panel).

To investigate if the interaction of maize phytochromes with ZmPIF3.1 and/or ZmPIF3.2 is modulated by the ratio of red and far-red light, we first cloned the full-length cDNAs for maize *PhyA2, PhyB1, PhyB2* and *PhyC1* into expression vectors. We then expressed and reconstituted full-length, ^35^S Met-labeled holophytochrome phyA2, phyB1, phyB2 and phyC1 with a phycocyanobilin chromophore, after the method of Ni *et al.* (1999). We then anchored the ^35^S-labeled maize and Arabidopsis PIF fusion proteins, fused to the yeast GAL4 activation domain (GAD), to Protein A-coated magnetic beads using a commercial GAD antibody. The binding of ZmPIF3.1 and ZmPIF3.2 to the maize phytochromes was assayed by irradiating *in vitro* translated protein mix with R or FR to convert the phytochrome into predominant Pfr or Pr forms, respectively, followed by co-immunoprecipitation (Fig. 2). In this assay, phyB1 in the Pfr form showed strong interaction with both PIF3.1 and PIF3.2 proteins, analogous to the behavior of Arabidopsis phyB (Ni *et al.*, 1999) (Fig. 2A). In contrast, phyA2 showed little if any binding, and phyC1 did not show any detectable binding to any of the PIFs regardless of illumination (Fig. 2A, B). Unexpectedly, phyB2 in either form also did not show any binding to either of the PIF3 homeologs (Fig. 2A).

### Mutation of four phyB1 residues to those in phyB2 removes detectable affinity for ZmPIF3s

**Fig 3.**
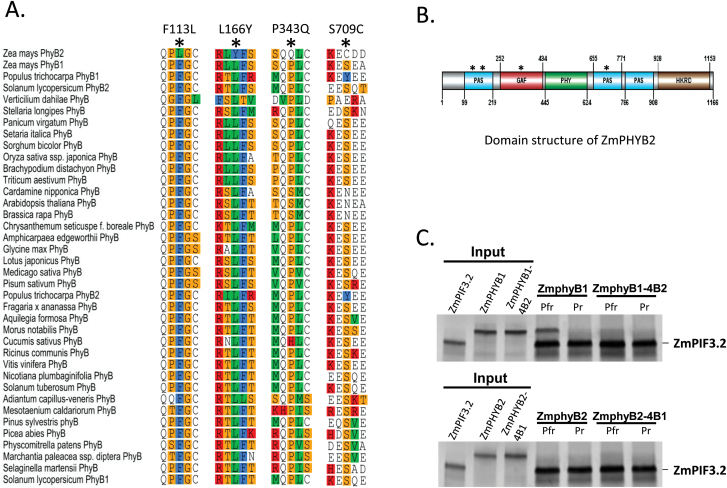
Identification of critical residues in maize phyB2 (ZmphyB2) potentially responsible for loss of PIF3 interaction. (A) Multiple alignment of selected conserved motifs from 38 PHYB apoprotein sequences from various angiosperms, mosses and ferns identifying four positions (F113L, L166Y, P343Q and S709C) where highly conserved amino acid residues in maize PHYB1 and PHYB from other organisms are substituted in maize PHYB2. (B) Domain map of maize PHYB2 showing the four potential candidate positions (marked with an asterisk) with respect to domain positioning. (C) Upper panel: in an *in vitro* co-immunoprecipitation experiment with ^35^S-labeled proteins, the PIF3.2 interaction with maize PhyB1 (ZmphyB1) in the active Pfr form was lost when these four conserved residues were substituted for those in maize phyB2 (ZmphyB2) (indicated by ZmphyB1-4B2). Lower panel: no detectable gain of interaction was observed when these four residues in maize PHYB2 were changed to mimic PHYB1 (indicated by ZmPHYB2-4B1). (This figure is available in colour at *JXB* online.)

Since maize PHYB1 and PHYB2 apoprotein sequences share more than 93% identity, the lack of phyB2 interaction with the ZmPIF3 proteins was unexpected. In order to ensure that phyB2 was not being misfolded or had otherwise not been produced in an active form, we used site-directed mutagenesis of the maize phyB1 protein to attempt to replicate the behavior of phyB2. A pairwise alignment of PHYB1 and PHYB2 apoproteins revealed 69 amino acid differences, including some insertion/deletion polymorphisms (Supplementary Fig. S5). To identify conserved residues likely to be responsible for interacting with PIF3, maize PHYB1 and PHYB2 protein sequences were compared to 36 different available PHYB sequences from various plants including angiosperms, mosses and ferns (Fig. 3A). Using the multiple sequence alignment of all 38 PHYB protein sequences, we identified four amino acid positions (113, 166, 343 and 709) in maize PHYB2 that carried a different amino acid residue to a highly conserved residue present in maize PHYB1 and other known PHYB sequences from a wide taxonomic range of plants (Fig. 3A). Positions 113 and 166 are part of the first PAS domain, position 343 is in the GAF domain and position 709 is in the second PAS domain of maize PHYB2 (Fig. 3B). Position 113 shows a conserved phenylalanine altered to leucine in PHYB2 (an aromatic to aliphatic side chain). A conserved leucine is altered to tyrosine (hydrophobic to hydrophilic) in PHYB2 at position 166. Position 343 is usually a proline, and occasionally a serine or histidine residue in other PHYBs. A glutamine residue at position 343 was not found in any other PHYB except ZmPHYB2. Similarly, at position 709, the conserved residue is primarily serine with some cases of aspargine and tyrosine, however ZmPHYB2 contains a cysteine residue.

In an experiment to determine whether the alteration of conserved residues in phyB1 could be responsible for the observed loss of holophytochrome phyB2 affinity to PIFs, the amino acid residues at these four candidate positions in ZmPHYB1 were changed to those in ZmPHYB2 using site-directed mutagenesis. The mutagenized chromophorylated holophytochrome was thus designated ZmphyB1-4B2. Immunoprecipitation using ZmPIF3.2 as bait and ZmphyB1-4B2 as prey revealed loss of affinity of phyB1-4B2 Pfr to PIF3.2, indicating that these residues are indeed necessary for the phy-PIF interaction (Fig. 3C, upper panel). In the reverse approach, we also checked if changing these residues in ZmPHYB2 was sufficient to confer PIF3 binding. ZmphyB2-4B1 (Pfr) did not show detectable binding to ZmPIF3.2 in the immunoprecipitation studies (Fig 3C, lower panel). Thus, these four amino acid residues in maize PHYB1/B2 seem to be required but not sufficient for PIF3 binding, and differences at other sites also contribute to the loss of affinity of phyB2 Pfr for PIFs.

### The interaction between phyB and PIF3 is conserved between maize and Arabidopsis

**Fig 4.**
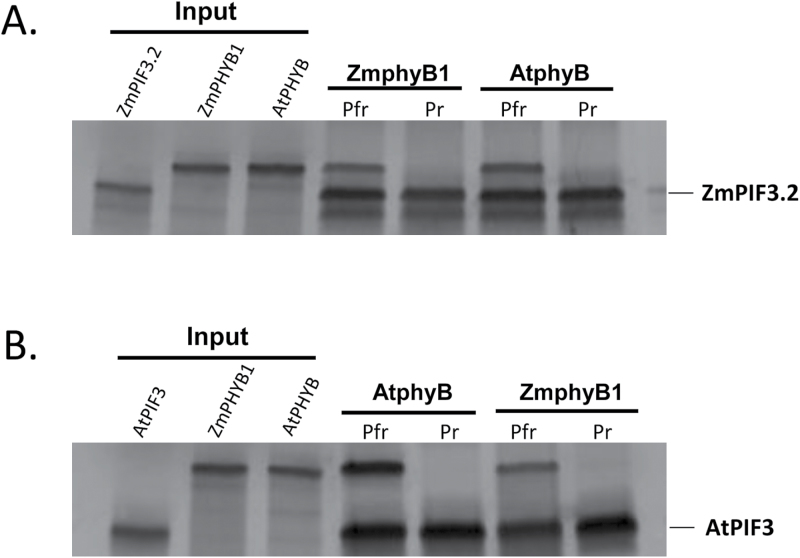
Immunoprecipitation assay showing *in vitro* interactions with PIF3 are conserved between the Pfr forms of maize and Arabidopsis PHYB. (A) Gel autoradiograph showing Arabidopsis phyB (AtphyB) in the Pfr form interacts with maize PIF3.2 (ZmPIF3.2) in a similar fashion to maize phyB1; (B) ZmphyB1(Pfr) interacts with AtPIF3. The ZmphyB1(Pfr)-ZmPIF3.2 and AtphyB(Pfr)-AtPIF3 interactions are positive controls.

Given the result that the recently diverged ZmphyB1 and ZmphyB2 behave differently in terms of PIF3 interaction, we set out to determine whether the PIF3:phyB interaction was conserved across greater evolutionary distances. Arabidopsis and maize PHYB proteins share a high level of amino acid identity (Supplementary Fig. S6) including the residues shown to be necessary for binding ZmPIF proteins in ZmPHYB1 that are altered in ZmPHYB2. Similarly, Arabidopsis and maize PIF3s possess substantial sequence-level conservation in the bHLH domain and APB/APA motifs (Fig. 1A, B). While there is little conservation outside of the bHLH domain and APB/APA motifs, the high level of conservation within the bHLH domains suggested that purifying selection may have acted on a phyB-PIF3 signaling interaction in Arabidopsis and maize that originated in a common ancestor of these two species. If the structural basis of the phy-PIF interaction were completely conserved, protein-protein interaction between Arabidopsis PIF3 and maize phyB, and vice versa, would be observed. The residues required for interaction between ZmphyB1 and PIF3 that were altered in ZmphyB2 were otherwise highly conserved, including between maize and Arabidopsis. If the protein-protein interaction of phyB with PIF3 is conserved between maize phyB1 and Arabidopsis phyB, this would imply that a molecular function conserved in phyBs since before the monocot-dicot divergence has been lost in ZmphyB2 more recently (after the allopolyploidy event creating the two *PhyB* genes in maize). To test this, we performed a targeted Y2H assay that showed C-terminal constructs of maize (Zm) PHYA2, PHYB1 and PHYB2 can interact with Arabidopsis (At) PIF3 (Supplementary Figs S2, S7). In the reverse experiment, the C-terminal domain of AtPHYB also showed interactions with both ZmPIF3.1 and ZmPIF3.2 (Supplementary Fig. S7C). Since full length, photoconvertible holophytochromes have significantly more complex structures, we produced full-length AtphyB holophytochrome using procedures and clones described by Ni *et al.* (1999). We then checked the interaction of ZmphyB1 holoprotein with AtPIF3 and AtphyB holoprotein with ZmPIF3.2 using immunoprecipitation after irradiation with R or FR. As shown in Fig. 4B, ZmphyB1 binds with AtPIF3 only in the Pfr form in a similar manner to AtphyB in the same experiment, and AtphyB (Pfr) also interacts with ZmPIF3.2 under these conditions (Fig. 4A). Thus, Arabidopsis phyB and maize phyB1, and Arabidopsis PIFs and maize PIFs can substitute for one another *in vitro* in the light-dependent interaction of the Pfr form of phyB family holoproteins with PIFs, but maize phyB2 cannot substitute for phyB1.

## Discussion

Our results indicate that maize has a family of bHLH proteins, with conserved domains present in the land plant lineages, and that these can be classified into subgroups similar to other higher plants (Toledo-Ortiz *et al.*, 2003; Hudson and Hudson, 2014, 2015). The maize VIIa/b subfamily contains seven sequences that likely have the capacity to bind active phytochrome, based on the presence of the APB domain. Notably, one of the maize PIFs was identified as an abundant transcript in an etiolated seedling cDNA library. This and a closely related homolog were shown to interact with phyB1. The bHLH domain and APB region of the PIF proteins are strongly conserved at the protein sequence level between Arabidopsis and maize, providing evidence that function is also conserved; however, sequence conservation outside these domains is much less strong. We also showed that as for Arabidopsis phyB, maize phyB1 Pfr has a much stronger affinity for PIFs than A or C type phytochromes, implying a conserved and distinct role for B-type phytochromes in high-affinity binding of Pfr to PIF3 orthologs. We did not observe readily detectable binding of ZmPIF3s to phyA under our conditions, despite the presence of a conserved APA domain in the protein sequence. This may reflect a lower affinity of the ZmPIF3s for ZmphyA to that previously observed between Arabidopsis phyA and PIF3, but may also be attributable to the limited ability of our experimental methods to detect a relatively weak interaction.

Our results from Y2H studies indicated that at least one part of the PHY and PIF signaling system is conserved at the level of the structure and function of the protein surfaces interacting between the C terminal portion of Arabidopsis and maize phyB and the PIF3 homologs of both species. We then tested the light-dependent interaction of the full-length phyB holoproteins with PIFs, and concluded that the Pfr form of maize phyB1 can interact with Arabidopsis PIF3, and the Pfr form of Arabidopsis phyB can interact with ZmPIF proteins, with similar affinity to the interaction shown by proteins from the same species. However, interactions between maize phyB2 and PIFs were not observed, and we confirmed that this is a result of changes in the amino acid sequences that have occurred since the divergence of phyB2 from phyB1 by artificially making selected changes to conserved residues in phyB1. These results have implications described in the following sections.

### Conservation of phyB-PIF interactions

The phyB-PIF3 interaction is highly conserved at the level of structure and biochemistry between maize (a monocot) and Arabidopsis (a dicot), indicating that the phyB and PIF3 proteins in these two species are orthologous in the strictest sense. This critical signaling interaction is therefore likely to be conserved among most or all of the Magnoliophyta (angiosperms). We have been unable to find evidence for the existence of this interaction outside the Magnoliophyta, and the evolution of this mechanism may, speculatively, have been important in the global ecological success of flowering plants.

### Subfunctionalization of phyB1 and phyB2

The phyB-PIF3 interaction is not conserved between the ZmPIF3s and phyB2 from maize, even though the genes *PhyB1* and *PhyB2* are the products of a recent evolutionary duplication ~11 Mya (Gaut, 2001; Sheehan *et al.*, 2004). While we cannot rule out residual affinity for PIF3 in phyB2, it is reduced below detectability in our assay, and therefore if not eliminated is very much less. Therefore the evolutionary forces (most likely purifying selection) acting to maintain the interaction are likely acting on *PhyB1* but not acting on *PhyB2*. This provides further evidence, this time at the level of protein biochemical function, for subfunctionalization of phyB1 and phyB2. Despite greatly reduced or eliminated affinity for PIFs, phyB2 is clearly biologically active, as demonstrated by the notable early flowering phenotype of the *phyb2* mutant of maize and the more severe phenotype of the *phyb1 phyb2* double mutant than either single mutant. Therefore, phyB1 and phyB2 both remain functional, but have evolved different roles at the level of protein-protein interaction. This represents an evolutionarily recent example of subfunctionalization at the level of molecular function of a protein, in addition to the process of differential evolution of gene expression patterns as described by Sheehan *et al.* (2007).

### PIF-independent signaling

A naturally occurring *phyb2* allele (*phyB2*-F2) in maize has a greater effect on flowering time than the *phyb1* mutation of maize (*phyB1*-563) (Sheehan *et al.*, 2007), while the *phyb1* mutation has a much greater effect on seedling de-etiolation as measured by mesocotyl elongation. The phyB2 protein has either greatly reduced or completely lost binding activity to PIF3, and this is consistent with the relatively normal de-etiolation phenotype of the *phyb2* mutant since PIF3 functions in de-etiolation. However, phyB2 has not lost effectiveness in the signaling pathway leading to control of flowering time in maize. It is possible to explain this observation in terms of PIF-mediated signaling by means of high levels of phyB2 expression relative to phyB1 in cells important for flowering time determination, in addition to residual affinity of phyB2 for PIF proteins that is below our detection limit in this study. Another possible explanation would be the interaction of phyB2 with a newly evolved PIF that was not detected in our Y2H screens. However, another compelling explanation is that flowering time in maize is mediated by an alternative, PIF-independent signaling pathway of phytochromes.

### Maize phytochrome and shade avoidance

Many crops, including maize, achieve optimal yields when grown at high planting densities. Much of the significant increases in maize yields delivered by modern cultivars can be attributed to higher tolerance for crowding (Duvick, 1997) rather than an increase in yield on a per plant basis. It has been known for some time that maize displays a classic shade avoidance response (Smith, 1982, 1983) and the *phyB1* mutant displays constitutive shade avoidance typical of a *phyB* mutant (Sheehan *et al.*, 2007). However, the phenotype of the *phyB1* mutant of maize is not as severe as that of the *phyB* mutant of Arabidopsis in terms of deviation from normal morphology (Somers *et al.*, 1991). It is possible that the reason for the ready adaptation of maize to high density growth as a row crop is related to the relatively mild responses it shows to R:FR and high planting density, and that this, in turn, may be partly attributable to the presence of a functional phytochrome B that is not capable of PIF interaction, since PIFs are directly implicated in mediation of the shade avoidance response (Leivar *et al.*, 2012).

## Conclusions

The phyB1 protein of maize shares conserved PIF-interaction functions with Arabidopsis phyB, but not its relatively recently diverged relative, maize phyB2. This provides an example of recent subfunctionalization of plant genes at the protein function level, and provides potential clues as to why maize is highly adaptable to high density growth.

## Supplementary data

Supplementary data are available at *JXB* online.

Fig S1. Spectroradiometer readings of LED light sources used in darkroom experiments.

Fig S2. Targeted Y2H analysis of maize phyB and PIF3s.

Fig S3. Multiple alignment of 197 maize bHLH sequences.

Fig S4. Maximum likelihood analysis of maize bHLH domains.

Fig S5. Pairwise alignment of maize PHYB1 and PHYB2 proteins.

Fig S6. Multiple alignment of ZmPHYB1, ZmPHYB2 and AtPHYB.

Fig S7. Targeted Y2H analysis of Arabidopsis vs maize phyBs and PIF3s.

Table S1. Representative bHLH members from each phylogenetic subfamily in Arabidopsis, rice and *Physcomitrella.*

Table S2. Maize bHLHs with assigned ID numbers and other relevant information.

Table S3. Maize PIF family members.

Supplementary Data

## Abbreviations and phytochrome gene/protein nomenclature in maize:

*PhyX* and *phyX* refer to non-mutant and mutant allele: respectively
PHYX and phyX refer to apoprotein and holophytochrome (apoprotein+chromophore): respectively.
