# Supplementary material for: Evolutionary divergence of phytochrome protein function in Zea mays PIF3 signaling
Source: J Exp Bot. 2016 Jun 4;67(14):4231–40. doi: 10.1093/jxb/erw217 (PMC5301934; doi:10.1093/jxb/erw217)
Supplement: Supplementary Data [file supp_67_14_4231__index.html]

Evolutionary divergence of phytochrome protein function in Zea mays PIF3 signaling — Evolutionary divergence of phytochrome protein function in Zea mays PIF3 signaling — Supplementary Data 

# Evolutionary divergence of phytochrome protein function in *Zea mays* PIF3 signaling

## Supplementary Data

Data files

- supplementary\_figures\_S1\_S7\_Tables\_S1\_S3.pdf - Supplementary Data
- Supplementary\_Table\_S2.xlsx - Supplementary Data
