# Supplementary material for: Evolutionary divergence of phytochrome protein function in Zea mays PIF3 signaling
Source: J Exp Bot. 2016 Jun 4;67(14):4231–40. doi: 10.1093/jxb/erw217 (PMC5301934; doi:10.1093/jxb/erw217)
Supplement: Supplementary Data [file supp_erw217_supplementary_figures_S1_S7_Tables_S1_S3.pdf]

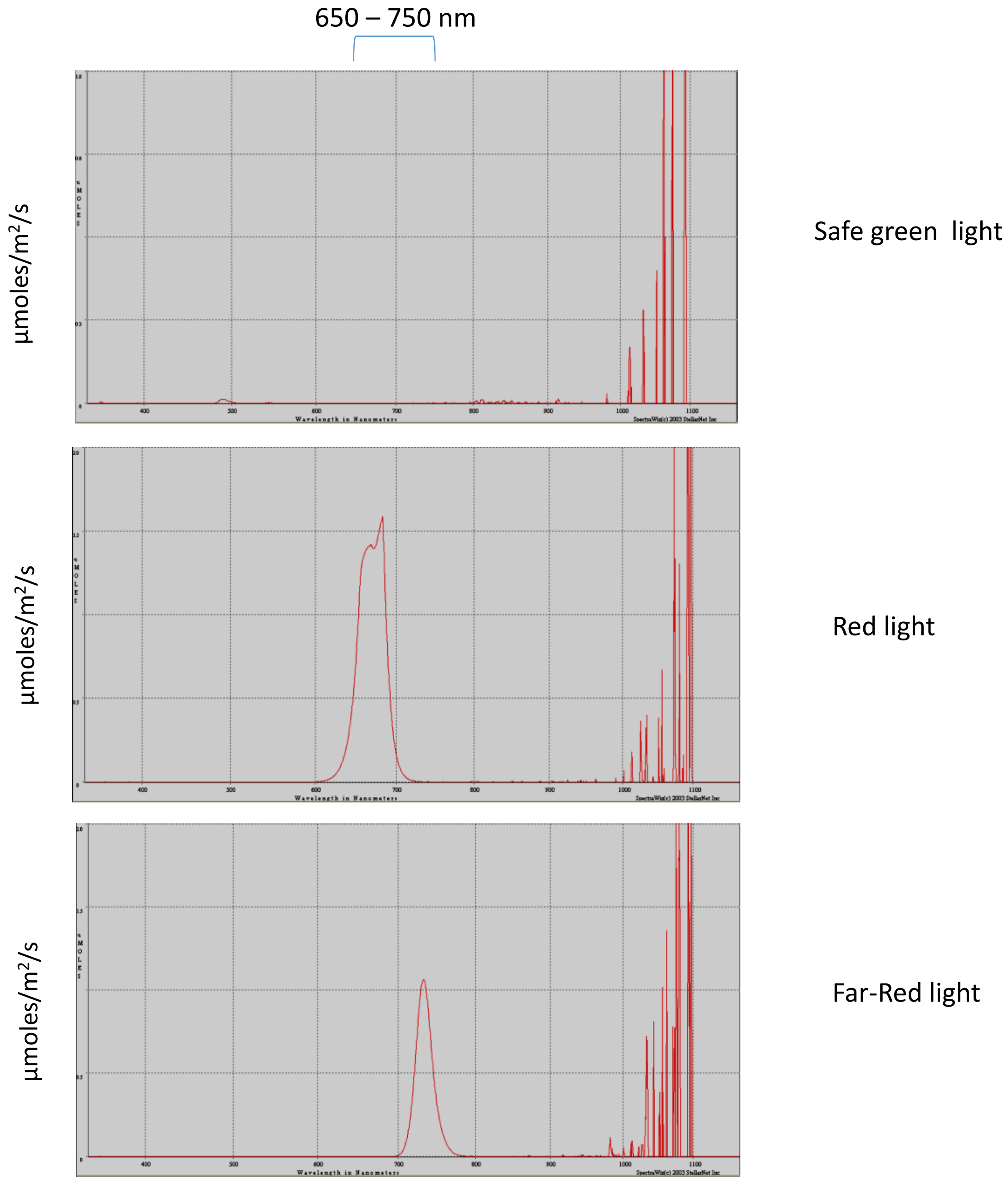

Fig S1: Spectroradiometer readings of LED light sources used in dark room experiments to photoconvert Pr and Pfr forms of phytochrome. Detector noise is present at wavelengths >1,000 nm, but the red and far-red bands do not overlap significantly.

A

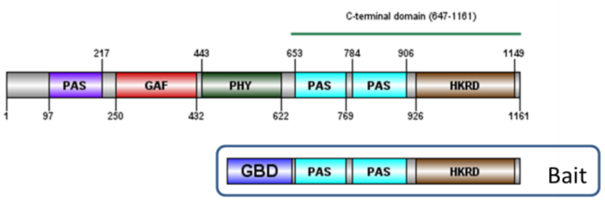

B

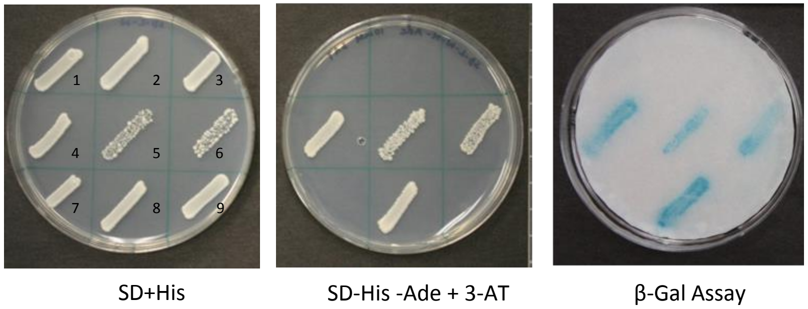

Section #	Bait	Prey
1	BD	AD:AtPIF3
2	BD	AD:ZmPIF3.2
3	BD	AD:ZmPIF3.1
4	BD:ZmPhyB1	AD:AtPIF3
5	BD:ZmPhyB1	AD:ZmPIF3.2
6	BD:ZmPhyB1	AD:ZmPIF3.1
7	BD	AD
8	BD:AtPHYB	AD:AtPIF3
9	BD:ZmPhyB1	AD

Fig S2: Targeted Yeast Two-Hybrid analysis. (A) Domain map of ZmPHYB1 (top) and the C-terminal region used as bait. (B) SD +His, SD-His-Ade+3AT plate and the β-Gal Assay showing the CTD of PHYB1 interacting with ZmPIF3.1 (#6), ZmPIF3.2 (#5) and AtPIF3 (#4). AtPhyB-CTD and AtPIF3 co-transformation (#8) was used as a position control.

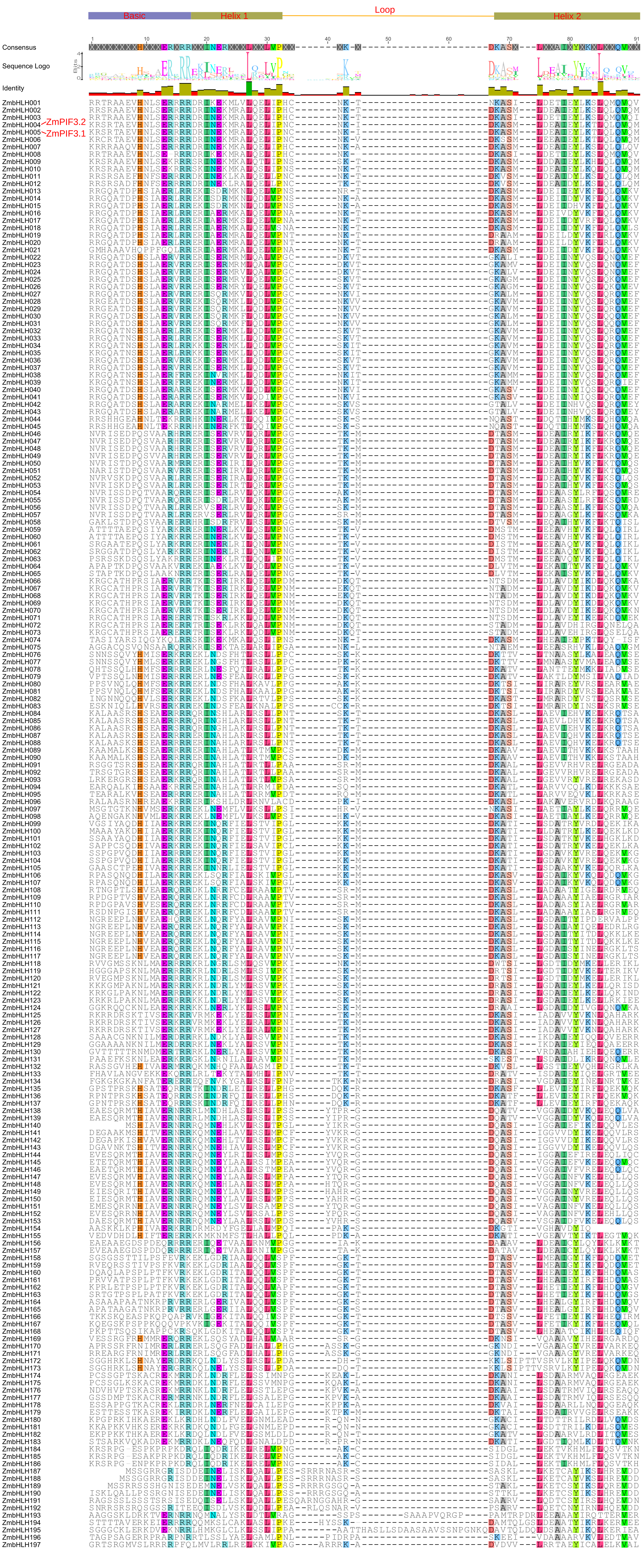

Fig S3: Multiple alignment of 197 maize bHLH sequences showing conserved residues, basic, helix-1, loop and helix-2 regions. ZmbHLH004 and ZmbHLH005 represent ZmPIF3.2 and ZmPIF3.1 respectively.

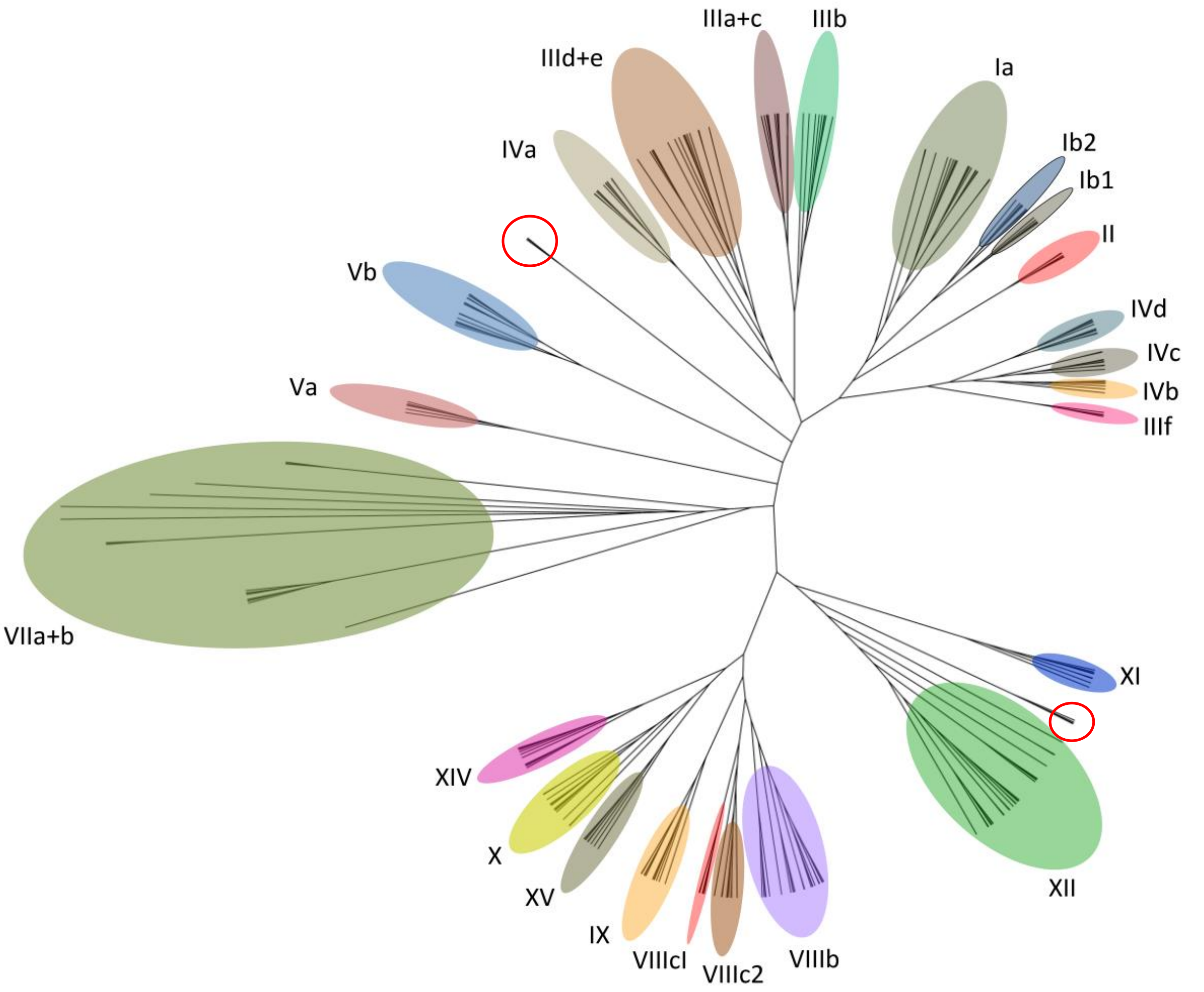

Fig S4: Cladogram of maximum likelihood analysis performed on the maize bHLH domains. Representative members from Arabidopsis, rice and *Physcomitrella* bHLH subfamilies were included in the multiple alignment for subfamily identification. Orphan members are marked by red circles.

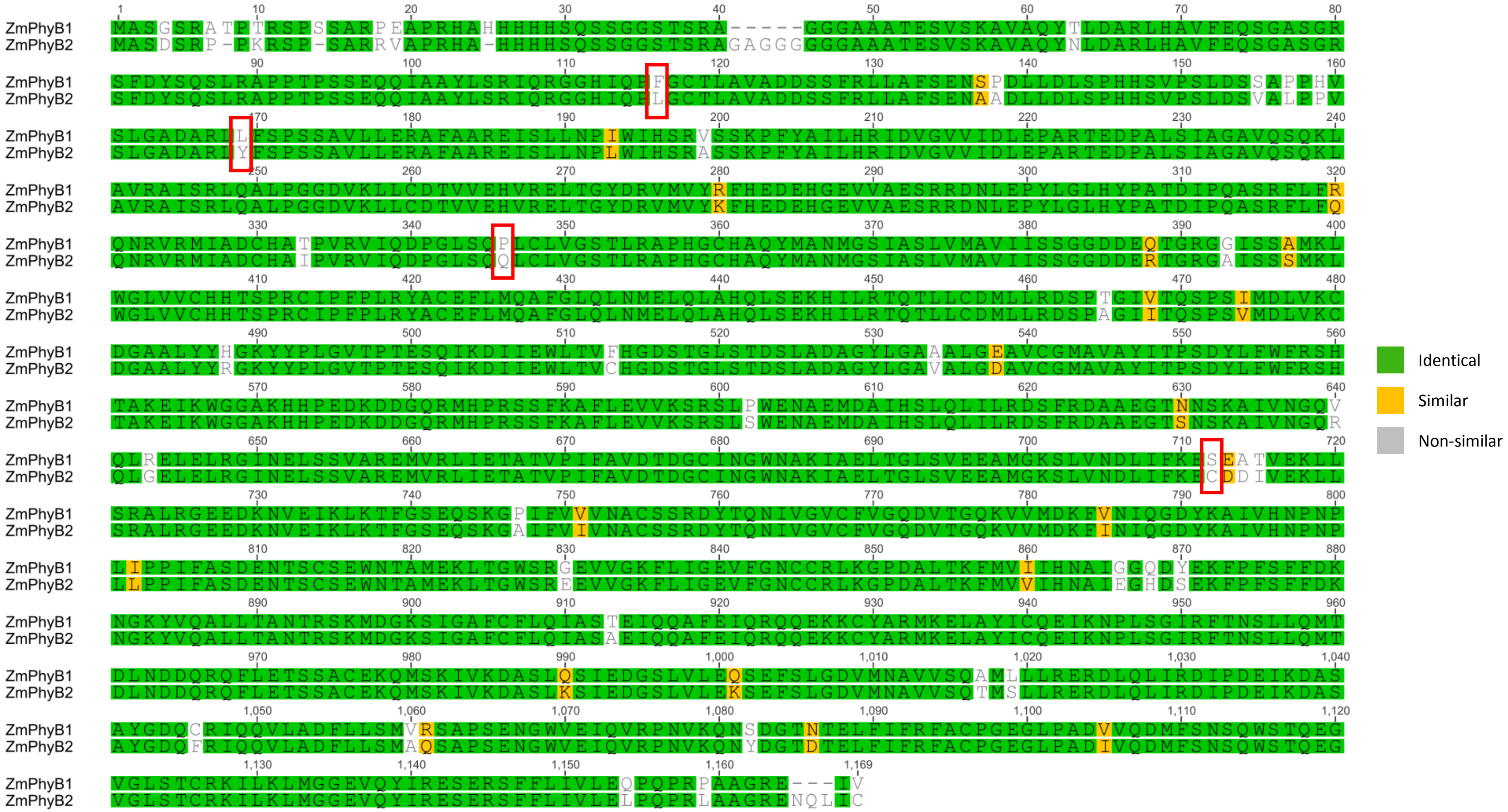

Fig S5: Pairwise alignment of maize PHYB1 and PHYB2 proteins showing positions with identical, similar and non-similar residues. A total of 69 differences were found including gaps. Positions marked with red boxes contain highly conserved amino acid residues in known PHYBs (see Fig 3A) that differ in ZmPHYB2. [Note: The amino acid numbering in this figure is for this pairwise alignment only].

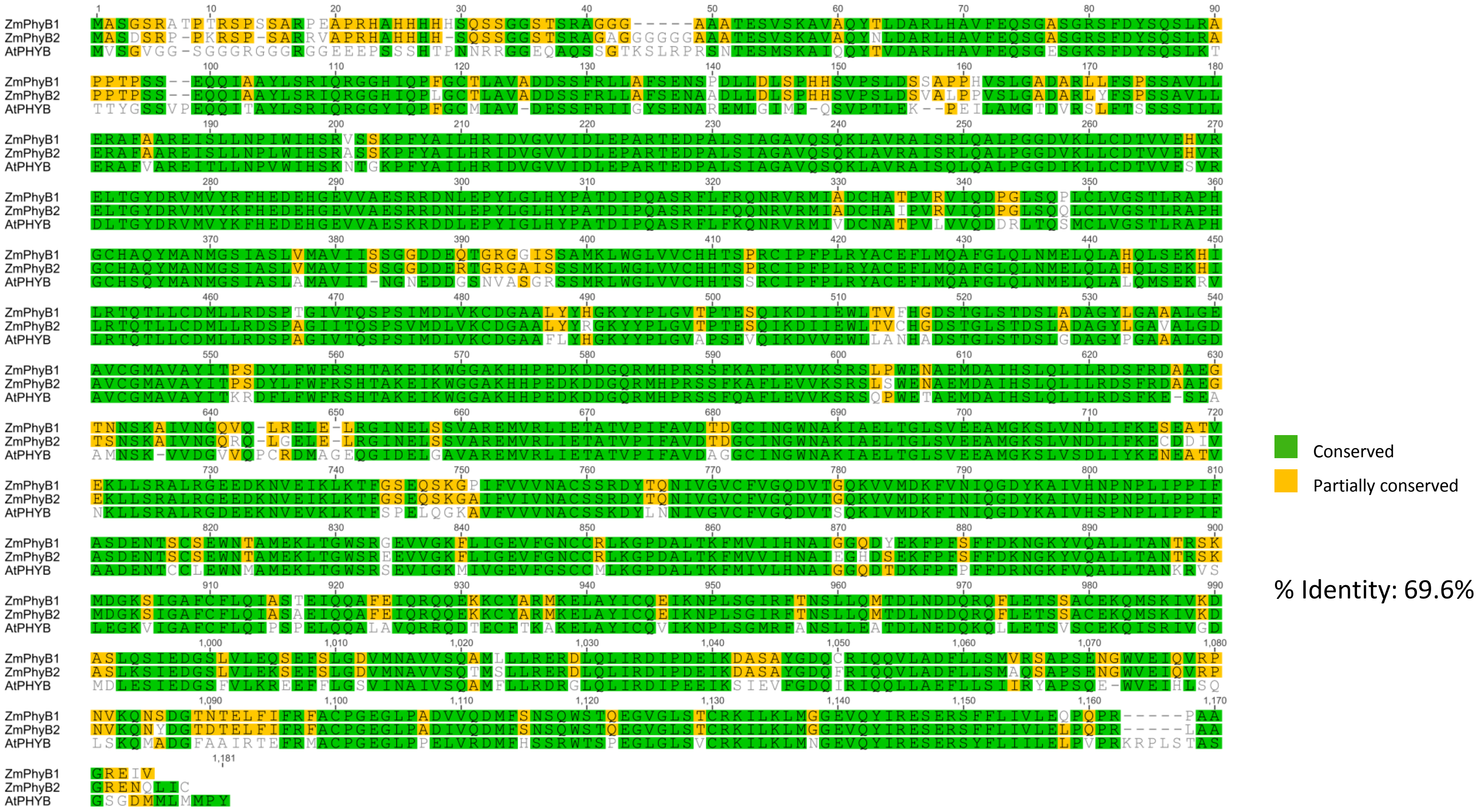

Fig S6: Multiple alignment of ZmPHYB1, ZmPHYB2 and AtPHYB showing high level of identity across the whole protein, and multiple nonconservative substitutions in ZmPHYB2.

A

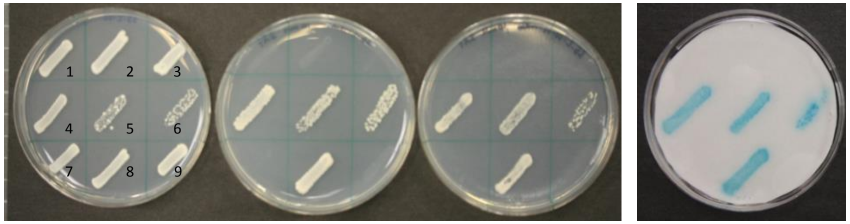

Section #	Bait	Prey
1	BD	AD:AtPIF3
2	BD	AD:ZmPIF3.2
3	BD	AD:ZmPIF3.1
4	BD:ZmPhyA2	AD:AtPIF3
5	BD:ZmPhyA2	AD:ZmPIF3.2
6	BD:ZmPhyA2	AD:ZmPIF3.1
7	BD	AD
8	BD:AtPHYB	AD:AtPIF3
9	BD:ZmPhyA2	AD

B

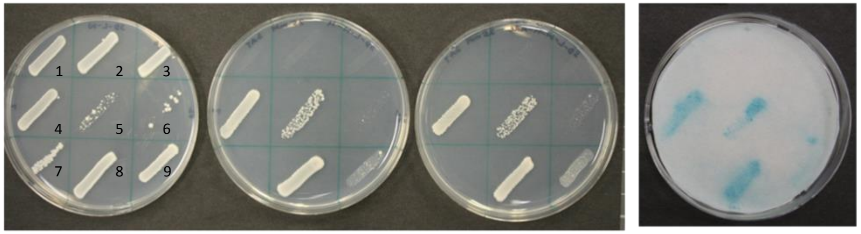

Section #	Bait	Prey
1	BD	AD:AtPIF3
2	BD	AD:ZmPIF3.2
3	BD	AD:ZmPIF3.1
4	BD:ZmPhyB2	AD:AtPIF3
5	BD:ZmPhyB2	AD:ZmPIF3.2
6	BD:ZmPhyB2	AD:ZmPIF3.1
7	BD	AD
8	BD:AtPHYB	AD:AtPIF3
9	BD:ZmPhyB2	AD

C

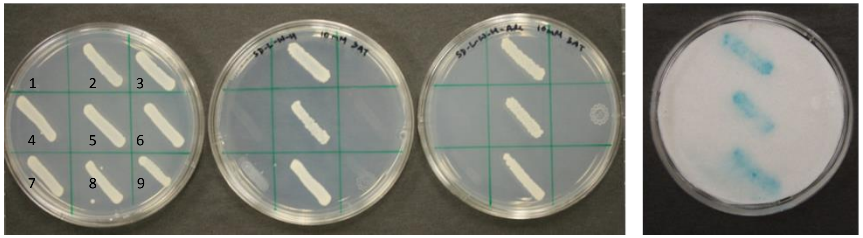

Section #	Bait	Prey
1	-	-
2	BD:AtPHYB	AD:ZmPIF3.1
3	BD	AD:ZmPIF3.1
4	BD	AD
5	BD:AtPHYB	AD:ZmPIF3.2
6	BD	AD:ZmPIF3.2
7	BD:AtPHYB	AD
8	BD:AtPHYB	AD:AtPIF3
9	BD	AD:AtPIF3

Fig S7: Targeted Yeast two hybrid analysis showing interaction between CTD of maize/Arabidopsis phytochromes with maize PIF3 homeologs and AtPIF3. Bait constructs used were as follows- (A) *ZmPhyA2*-CTD, (B) *ZmPhyB2*-CTD and (C) *AtPHYB*-CTD. Full length *ZmPIF3.1*, *ZmPIF3.2* and *AtPIF3* were used as prey. BD:*AtPHYB*-CTD-AD:AtPIF3 was used as positive control.

Sub group	Representatives
Ia	OsbHLH044, AtbHLH096, PpbHLH018
Ib(1)	OsbHLH144, AtbHLH095, PpbHLH037
Ib(2)	OsbHLH147, AtbHLH126, AtbHLH100
II	OsbHLH141, AtbHLH010, PpbHLH092
IIIa+c	OsbHLH006, AtbHLH029, PpbHLH041
IIIb	OsbHLH001, AtbHLH061, PpbHLH021
IIId+e	OsbHLH008, AtbHLH003, PpbHLH002
IIIf	OsbHLH012, AtbHLH012, PpbHLH004
Iva	OsbHLH018, AtbHLH025, PpbHLH022
Ivb	OsbHLH061, AtbHLH121, PpbHLH039
Ivc	OsbHLH057, AtbHLH115, PpbHLH014
Ivd	OsbHLH024, AtbHLH041, PpbHLH005
IX	OsbHLH109, AtbHLH128, PpbHLH006
Va	OsbHLH031, AtbHLH141, PpbHLH046
Vb	OsbHLH035, AtbHLH030
VIIa+b	OsbHLH101, AtbHLH008, AtbHLH026, PpbHLH009
VIIIa	OsbHLH178, AtbHLH117, PpbHLH074
VIIIb	OsbHLH117, AtbHLH140, PpbHLH026
VIIIc(1)	OsbHLH125, AtbHLH086, PpbHLH033
VIIIc(2)	OsbHLH128, AtbHLH054, AtbHLH084, PpbHLH028
X	OsbHLH065, AtbHLH110
XI	OsbHLH096, AtbHLH066, PpbHLH012
XII	OsbHLH079, AtbHLH078, PpbHLH001
XIII	OsbHLH149, AtbHLH156
XIV	OsbHLH138, AtbHLH142
XV	OsbHLH153, AtbHLH135

Table S1: Representative bHLH members from each phylogenetic subfamily in Arabidopsis, rice and *Physcomitrella* that were used in multiple sequence alignment along with the ZmbHLH sequences for bHLH subfamily classification in maize (Pires and Dolan, 2010).

Maize PIF subfamily

Gene Id	bHLH #	Annotation	Chr #	APB Motif	APA Motif	Rice Ortholog
GRMZM2G165042	ZmbHLH002		1	Y		LOC_Os03g43810 (PIL12)
GRMZM2G065374	ZmbHLH003	PIF5 Like	1	Y		LOC_Os03g56950.1 (OsPIL1/PIL13)
GRMZM2G016756	ZmbHLH008	PIF5 Like	5	Y		LOC_Os03g56950.1 (OsPIL1/PIL13)
GRMZM2G387528	ZmbHLH004	*PIF3.2* (this study)	8	Y	Y	LOC_Os05g04740 (OsPIL16)
GRMZM2G115960	ZmbHLH005	*PIF3.1* (this study)	3	Y	Y	LOC_Os05g04740 (OsPIL16)
GRMZM2G062541	ZmbHLH006	PIF1 Like	10	Y	Y	LOC_Os05g04740 (OsPIL16)
GRMZM5G865967	ZmbHLH007		1	Y		LOC_Os12g41650 (OSPIL11)

Table S3: The predicted maize PIF family contains at least 7 members. Genes highlighted in red and blue colors are homeologous pairs. The PIF3 annotation is based on experimental data presented elsewhere in this study. PIF5-like genes are annotated based on BLAST hits to Arabidopsis as well as functional studies performed on rice orthologs (Todaka *et al.*, 2012). Due to the presence of an APA motif detected using a Hidden Markov Model, GRMZM2G062541 was assigned an annotation of “PIF1 like”. Orthology of other maize PIFs could not be determined with any confidence.
